# Editorial: Medical knowledge-assisted machine learning technologies in individualized medicine

**DOI:** 10.3389/fmolb.2023.1167730

**Published:** 2023-03-24

**Authors:** Feng Gao, William C. Cho, Xin Gao, Wei Wang

**Affiliations:** ^1^ Department of Colorectal Surgery, Department of General Surgery, The Sixth Affiliated Hospital, Sun Yat-sen University, Guangzhou, China; ^2^ Shanghai Artificial Intelligence Laboratory, Shanghai, China; ^3^ Department of Clinical Oncology, Queen Elizabeth Hospital, Kowloon, Hong Kong SAR, China; ^4^ Computational Bioscience Research Center, King Abdullah University of Science and Technology (KAUST), Thuwal, Saudi Arabia; ^5^ Computer, Electrical and Mathematical Sciences and Engineering Division, King Abdullah University of Science and Technology (KAUST), Thuwal, Saudi Arabia; ^6^ Department of Pathology, First Affiliated Hospital of Anhui Medical University, Hefei, China

**Keywords:** individualized medicine, machine learning, medical knowledge, computational biology, medical image analysis, biomedical data analysis

Machine learning (ML), a powerful tool for mining quantitative disease features in large quantities of medical data, is arguably the most important method for extracting, integrating, and modeling patient-specific and disease-specific factors for individualized medicine. ML can provide clinical decision support for disease diagnosis, prognosis, and treatment in practice. In reality, there is no universal ML model or solution for all medical problems and clinical circumstances (Musolf et al., 2022), since both the fundamental biological mechanisms and the modality of the clinically interested medical data differ from disease to disease. In other words, biological and medical knowledge of the specific domain is essential for the development of ML models for the corresponding medical problems (You et al., 2022). For data scientists with rich modeling experience and limited medical background knowledge, considering even the simplest basic medical knowledge will significantly refine the structure of the ML framework and the process of model training, offering an opportunity for the establishment of ML models with higher performance and better interpretability (Chen et al., 2022). From another aspect, for biomedical researchers of different specialties, effectively incorporating ML models with clinical information and biological knowledge will lead to further insight into the pathogeneses of different diseases and new biomedical findings (Jin et al., 2021; Eloranta and Boman, 2022), which may translate into new drugs or therapeutic strategies for precision medicine. This Research Topic collects some of the contributions from both sides, providing insights into the medical knowledge-assisted ML studies for disease diagnosis, prognosis, and individualized treatment management.

Currently, the ML technique is more and more adopted in the field of genomics analysis. Albaradei et al. presented a pan-cancer model that takes the gene expression profile of the primary site and predicts whether and where the metastasis has occurred. According to previous studies, metastasis does not occur randomly, noting that the relationship between the metastasis clusters/cells and the specific remote organ is similar to the relationship between the seed and soil (Yuan et al., 2019). Following the “seed and soil” hypothesis, Albaradei et al. identified metastasis-related primary tumor transcriptome features of the common metastasis target organs, such as the lung, liver, bone, and brain. Incorporating the metastasis expression features for common metastasis target organs, the established model of this study perform well in pinpointing the metastasis site, aiding decision-making in clinical practice. In another study, Nath et al. developed an integrative ML model predicting everolimus treatment response based on *in vitro* experimental results and gene expression data of a neoadjuvant everolimus therapy cohort. Hormonal therapy is the mainstay of the treatment for estrogen receptor-positive breast cancer, but many estrogen receptor-positive cases resist endocrine therapy (Hanker et al., 2020). The ML model established by Nath et al. integrated experimentally verified everolimus response signatures with features generated by *in silico* analysis of transcriptome data in the clinical cohort, providing accurate everolimus response prediction for individualized breast cancer treatment.

To advance ML-aided precision medicine, this Research Topic also discusses studies of medical images. Images of computer tomography (CT) are vital for the diagnosis, classification, and risk evaluation of intracerebral hemorrhage (ICH) (Li et al., 2021). Nijiati et al. pointed out that one of the most important characteristics of the ICH CT images is the destruction of the symmetrical structure of the brain tissue. With this prior anatomical knowledge, the authors integrated a transformer-based framework to capture the long-distance symmetric information in their Sym-TransNet model, achieving higher ICH lesion segmentation performance than the existing methods. In addition to ICH, CT scans also play an important role in clinical oncology. Li et al. proposed a multi-size convolutional neural network-based model for colorectal cancer recurrence prediction based on CT images. With the help of the human experts’ annotation of CT images, the model crop and magnify the inputted CT images at different magnifications to extract radiological features of multiple levels, turning each CT image into a series of images containing both full-image level information and detailed tumoral level information imitating the CT scan reading behavior of the human experts. The model succeeds in predicting patients with high recurrence risk, providing a reliable tool for individualized prognosis evaluation. Except for CT, ultrasonography also plays a part in clinical oncology. Zhu et al. developed a diagnosis model for the evaluation of the malignant degrees of renal tumors based on ultrasound images. The model extract features from both B-mode ultrasound and contrast-enhanced ultrasound-mode images that are annotated by senior radiologists, reaching an expert-level accuracy in differentiating benign cases from malignant cases.

In conclusion, the integration of medical knowledge into ML technology has been regarded as a powerful computer-aided tool for individualized medicine. Aiming at providing a broad view of the fascinating development in the field, this Research Topic collected articles that gave some insights to make good use of ML technology in a clever way to deal with a variety of clinical or biological problems. We envision this Research Topic of articles will provide insights for researchers interested in medical data science, speed up the development of medical knowledge-aided ML methods, and reinforce the clinical applications of the ML models for precision medicine.
